# Supplementary material for: Anti-Inflammatory Activity of Glycolipids and a Polyunsaturated Fatty Acid Methyl Ester Isolated from the Marine Dinoflagellate Karenia mikimotoi
Source: Mar Drugs. 2020 Feb 27;18(3):138. doi: 10.3390/md18030138 (PMC7143331; doi:10.3390/md18030138)

# Supporting Information

## Glycolipids and a Polyunsaturated Fatty Acid Methyl Ester Isolated from the Marine Dinoflagellate *Karenia mikimotoi*

Alain S. Leutou, Jennifer R. McCall, Bob York, Rajeshwar R. Govindapur and Andrea J. Bourdelais *

Center for Marine Science, University of North Carolina at Wilmington, 5600 Marvin K. Moss Lane, USA; leutoualain@yahoo.fr, (A.S.L.); mccalljr@uncw.edu (J.R.M.); yorkra@uncw.edu (B.Y.); govindapurr@uncw.edu (R.R.G.)

*A. J. B. Tel: +1-910-431-3645. E-mail: bourdelaisa@uncw.edu.

# Table of Contents


**Figure S1.** $^{1}H$ NMR Spectrum (500 MHz) of Monogalactosyldiacylglycerol (**1**) in $CD_3OD$ ........ **S3**

**Figure S2.** $^{13}C$ NMR Spectrum (125 MHz) of Monogalactosyldiacylglycerol (**1**) in $CD_3OD$ ........ **S4**

**Figure S3.** DEPT-135 (125 MHz) of Monogalactosyldiacylglycerol (**1**) in $CD_3OD$ ........ **S5**

**Fiure S4.** COSY Spectra (500 MHz) of Monogalactosyldiacylglycerol (**1**) in $CD_3OD$ ........ **S6**

**Figure S5.** HSQC Spectra (500 MHz) of of Monogalactosyldiacylglycerol (**1**) in $CD_3OD$ ........ **S7**

**Figure S6.** HMBC Spectra (500 MHz) of of Monogalactosyldiacylglycerol (**1**) in $CD_3OD$ ........ **S8**

**Figure S7.** HRMSESITOF of Monogalactosyldiacylglycerol (**1**) ........ **S9**

**Figure S8.** IR Spectra of Monogalactosyldiacylglycerol (**1**) ........ **S10**

**Figure S9.** Fragmentation ESI-MS of the monogalactosyldiacylglycerol (**1**) ........ **S11**

**Figure S10.** $^{1}H$ NMR Spectrum (500 MHz) of Monogalactosylmonoacylglycerol (**2**) in $CD_3OD$ ........ **S12**

**Figure S11.** $^{13}C$ NMR Spectrum (125 MHz) of Monogalactosylmonoacylglycerol (**2**) in $CD_3OD$ ........ **S13**

**Figure S12.** HRMSESITOF of Monogalactosylmonoacylglycerol (**2**) ........ **S14**

**Figure S13.** $^{1}H$ NMR Spectrum (500 MHz) of Polyunsaturated fatty acid methyl ester (**3**) in $CD_3OD$ ........ **S15**

**Figure S14.** $^{13}C$ NMR Spectrum (125 MHz) of Polyunsaturated fatty acid methyl ester (**3**) in $CD_3OD$ ........ **S16**

**Figure S15.** HRMSESITOF of Polyunsaturated fatty acid methyl ester (**3**) ........ **S17**

**Figure S1.** $^1$H NMR Spectrum (500 MHz) of Monogalactosyldiacylglycerol (**1**) in $CD_3OD$

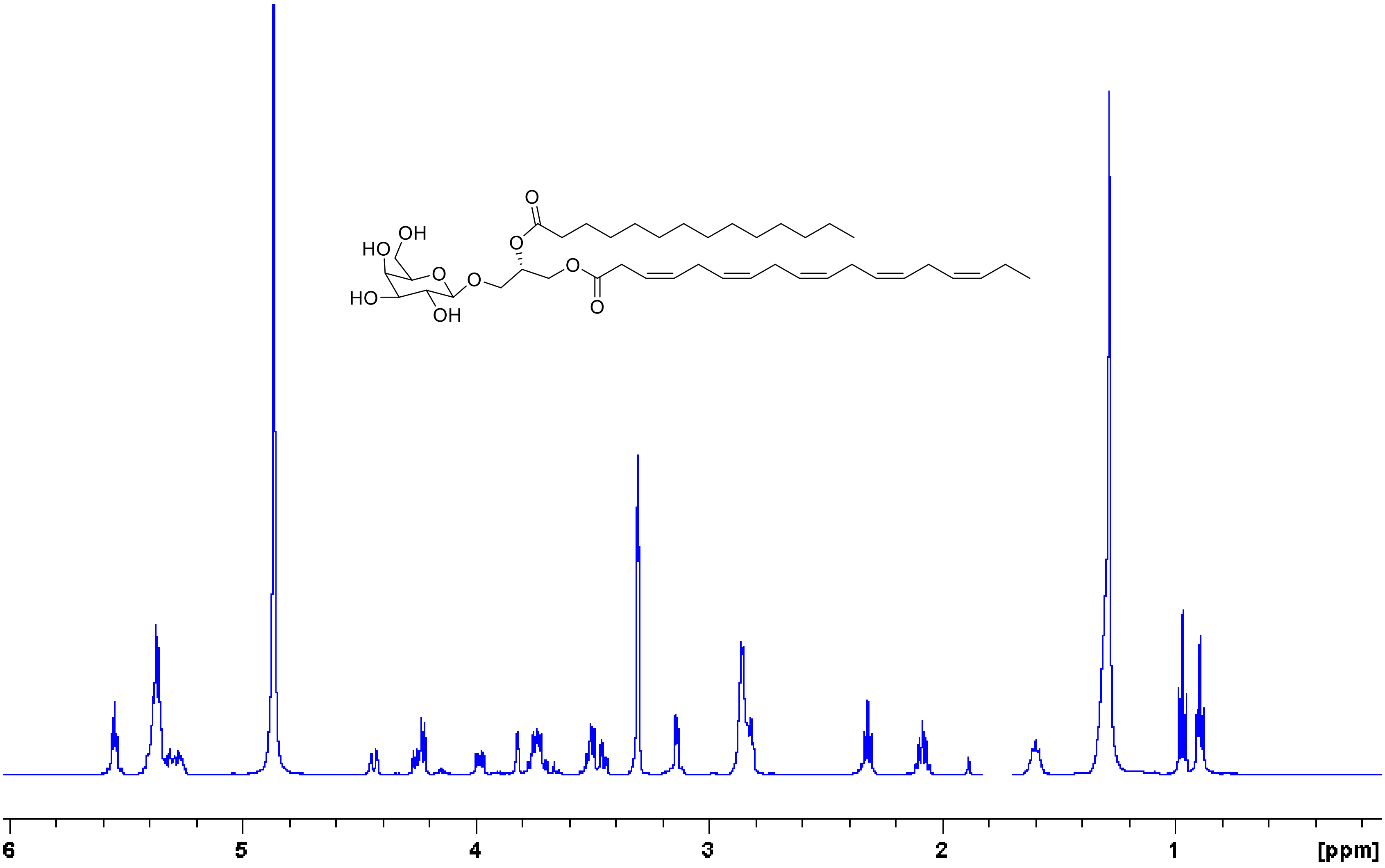

**Figure S2.** $^{13}C$ NMR Spectrum (125 MHz) of Monogalactosyldiacylglycerol (**1**) in $CD_3OD$

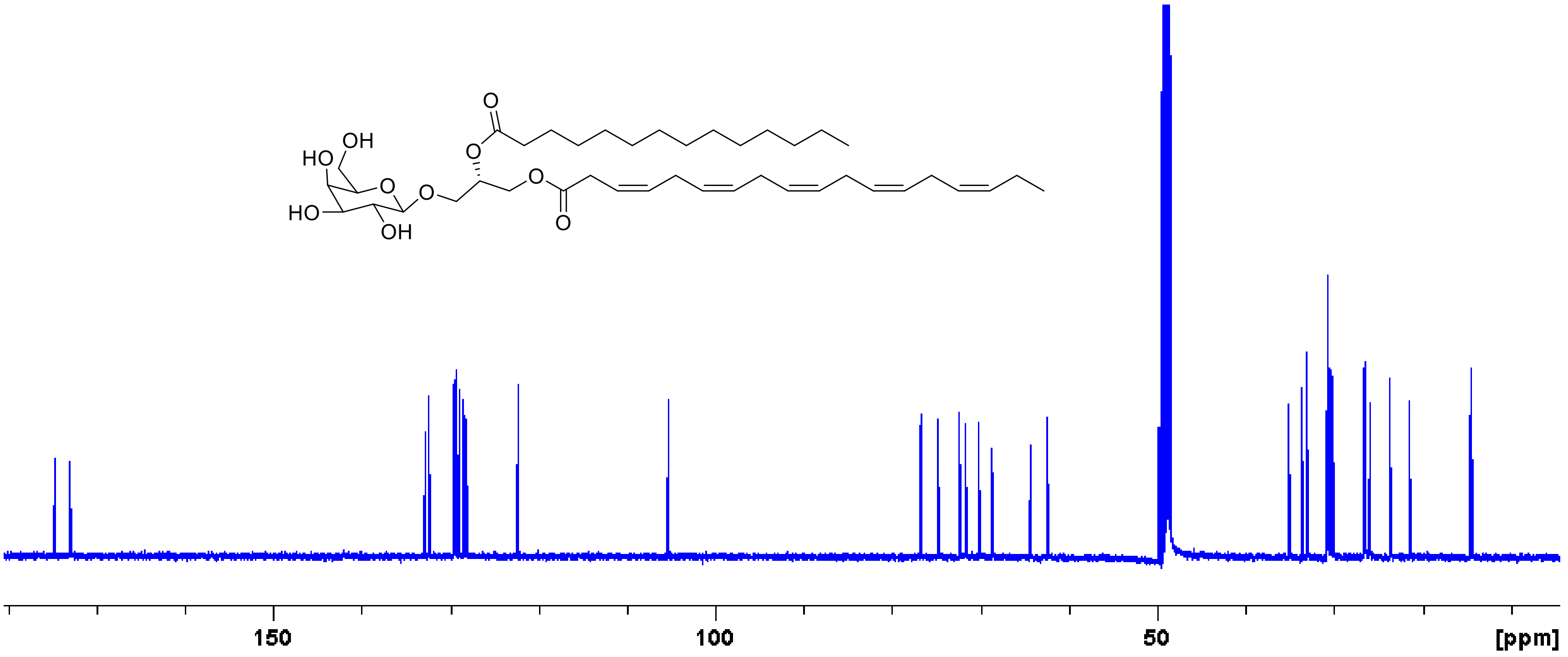

**Figure S3.** DEPT-135 (125 MHz) of Monogalactosyldiacylglycerol (**1**) in $CD_3OD$

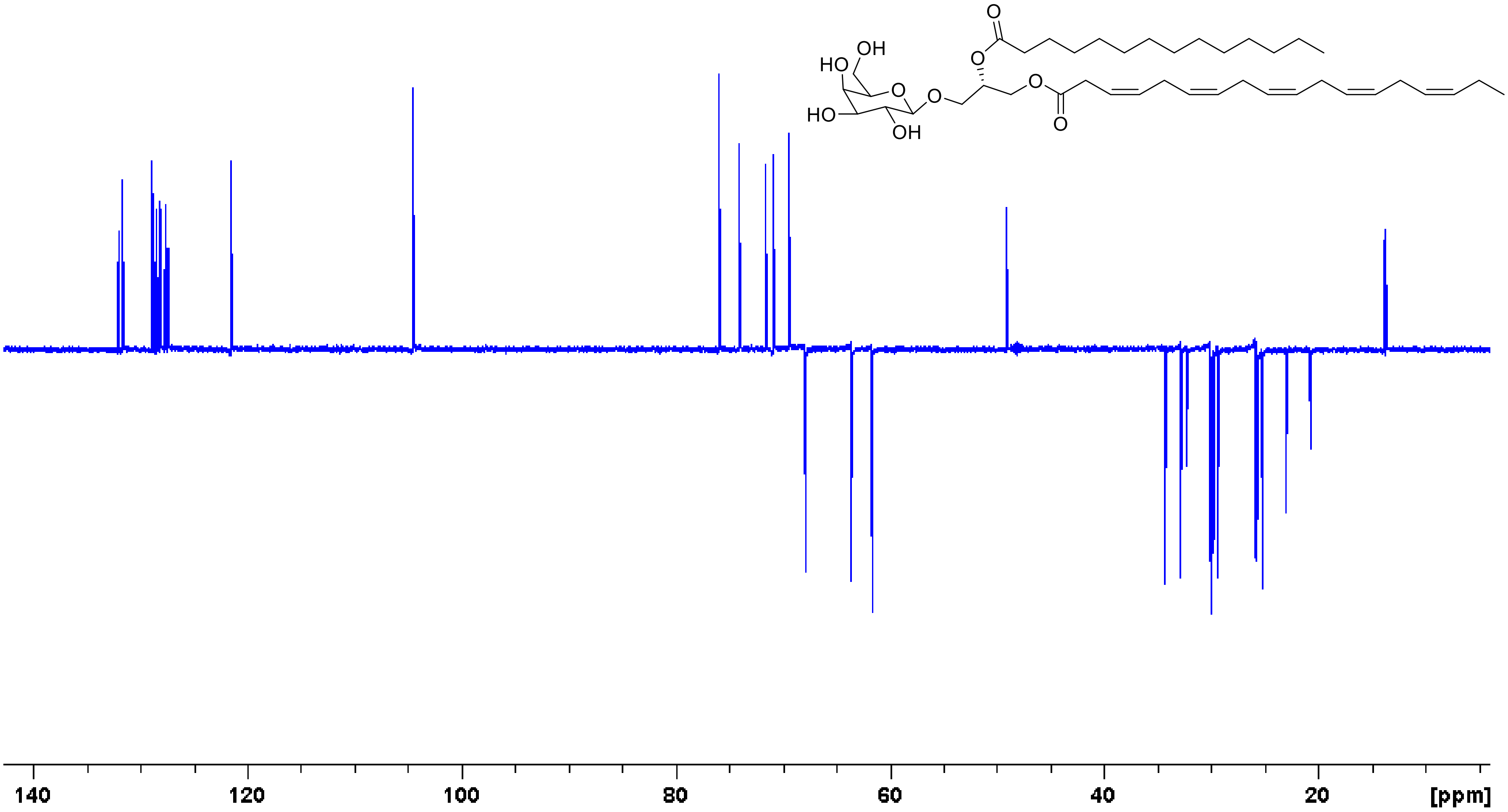

**Figure S4.** COSY Spectra (500 MHz) of Monogalactosyldiacylglycerol (**1**) in $CD_3OD$

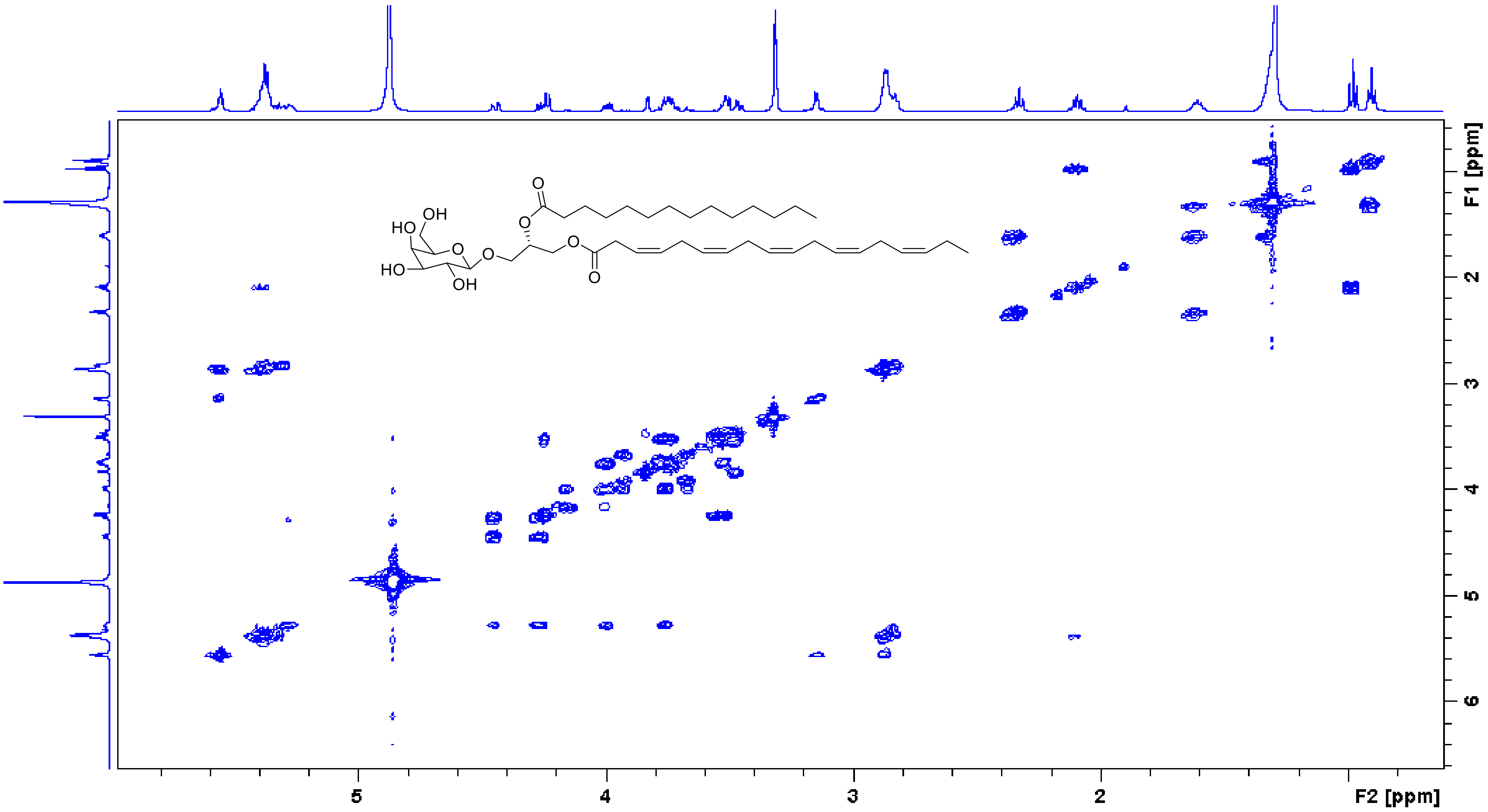

**Figure S5.** HSQC Spectra (500 MHz) of of Monogalactosyldiacylglycerol (**1**) in $CD_3OD$

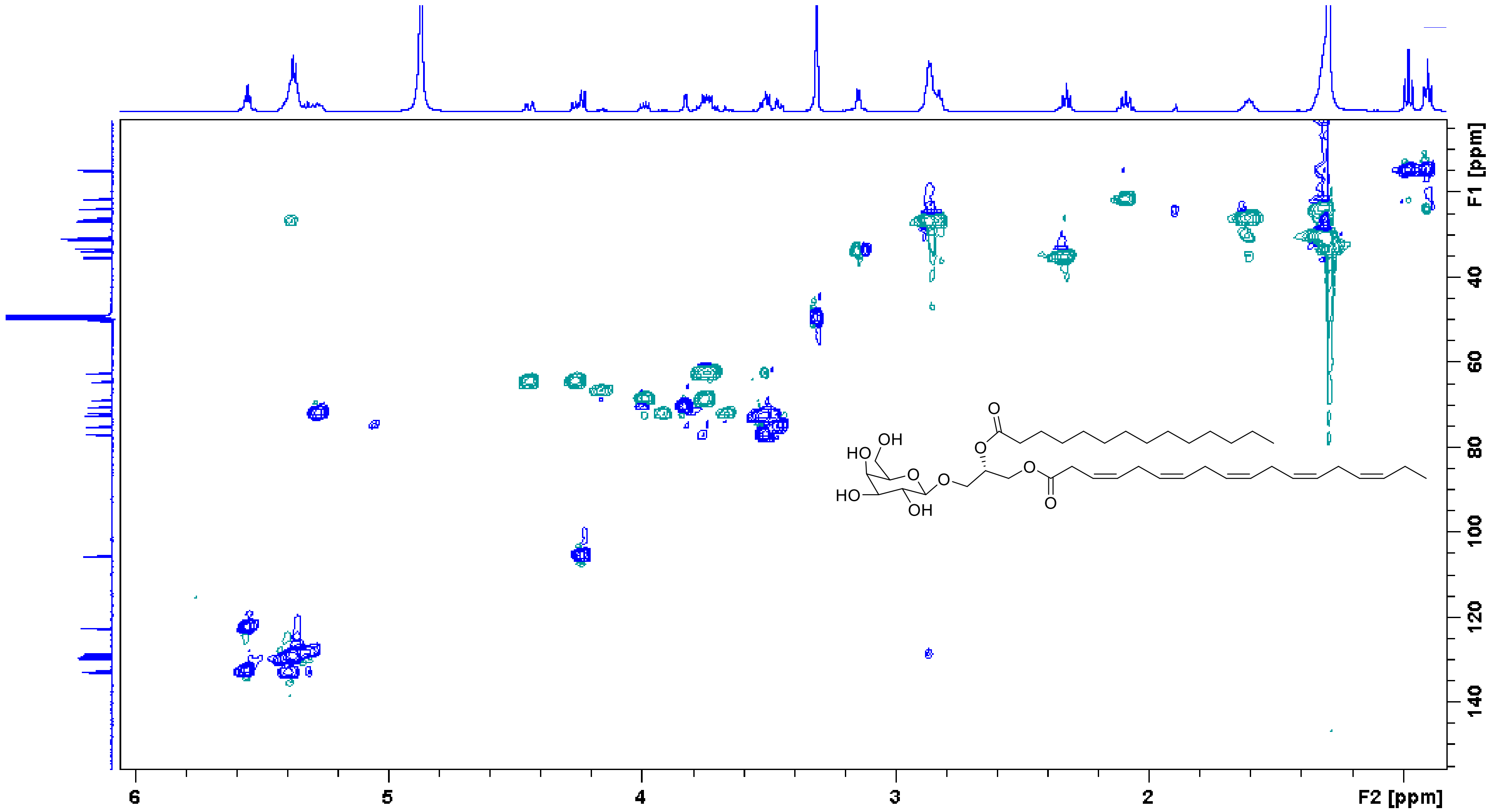

**Figure S6.** HMBC Spectra (500 MHz) of of Monogalactosyldiacylglycerol (**1**) in $CD_3OD$

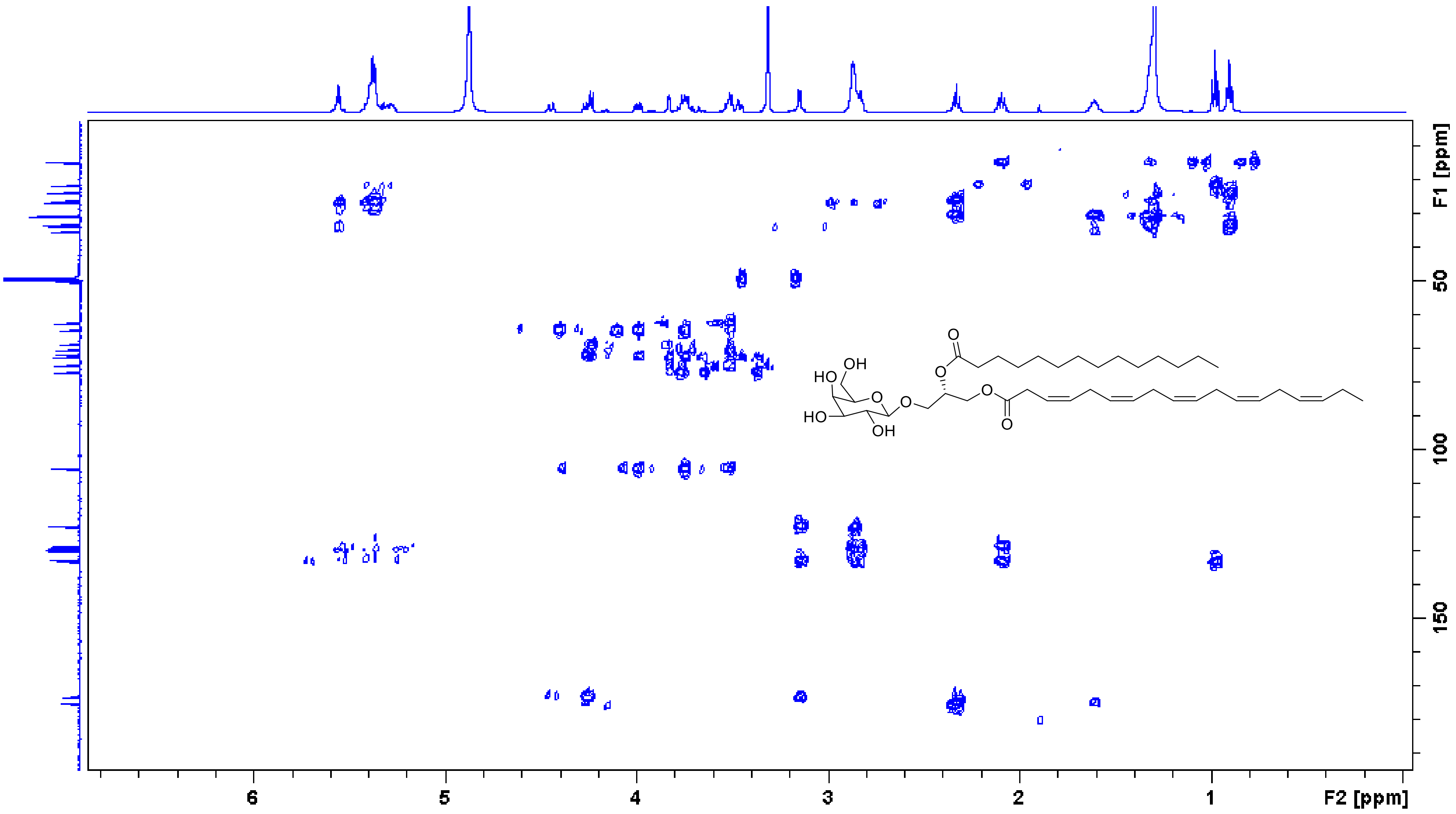

**Figure S7.** HRMSESITOF of Monogalactosyldiacylglycerol (**1**)

**Single Mass Analysis**
Tolerance = 4.0 PPM / DBE: min = 0.0, max = 50.0
Element prediction: Off
Number of isotope peaks used for i-FIT = 3
Monoisotopic Mass, Even Electron Ions
315 formula(e) evaluated with 2 results within limits (all results (up to 1000) for each mass)
Elements Used:

| Mass | Calc. Mass | mDa | PPM | DBE | Formula | i-FIT | i-FIT Norm | Fit Conf % | C | H | O | Na |
|---|---|---|---|---|---|---|---|---|---|---|---|---|
| 743.4727 | 743.4734 | -0.7 | -0.9 | 10.5 | C43 H67 O10 | 444.9 | 1.606 | 20.07 | 43 | 67 | 10 | |
| | 743.4710 | 1.7 | 2.3 | 7.5 | C41 H68 O10 Na | 443.5 | 0.224 | 79.93 | 41 | 68 | 10 | 1 |

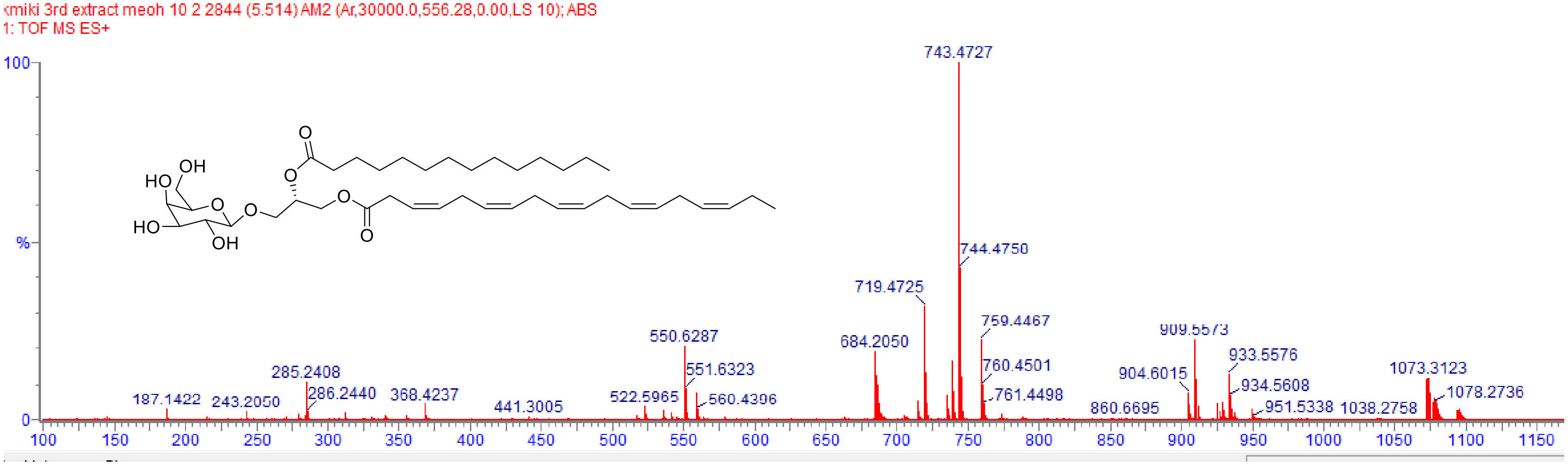

**Figure S8.** IR Spectra of Monogalactosyldiacylglycerol (**1**)

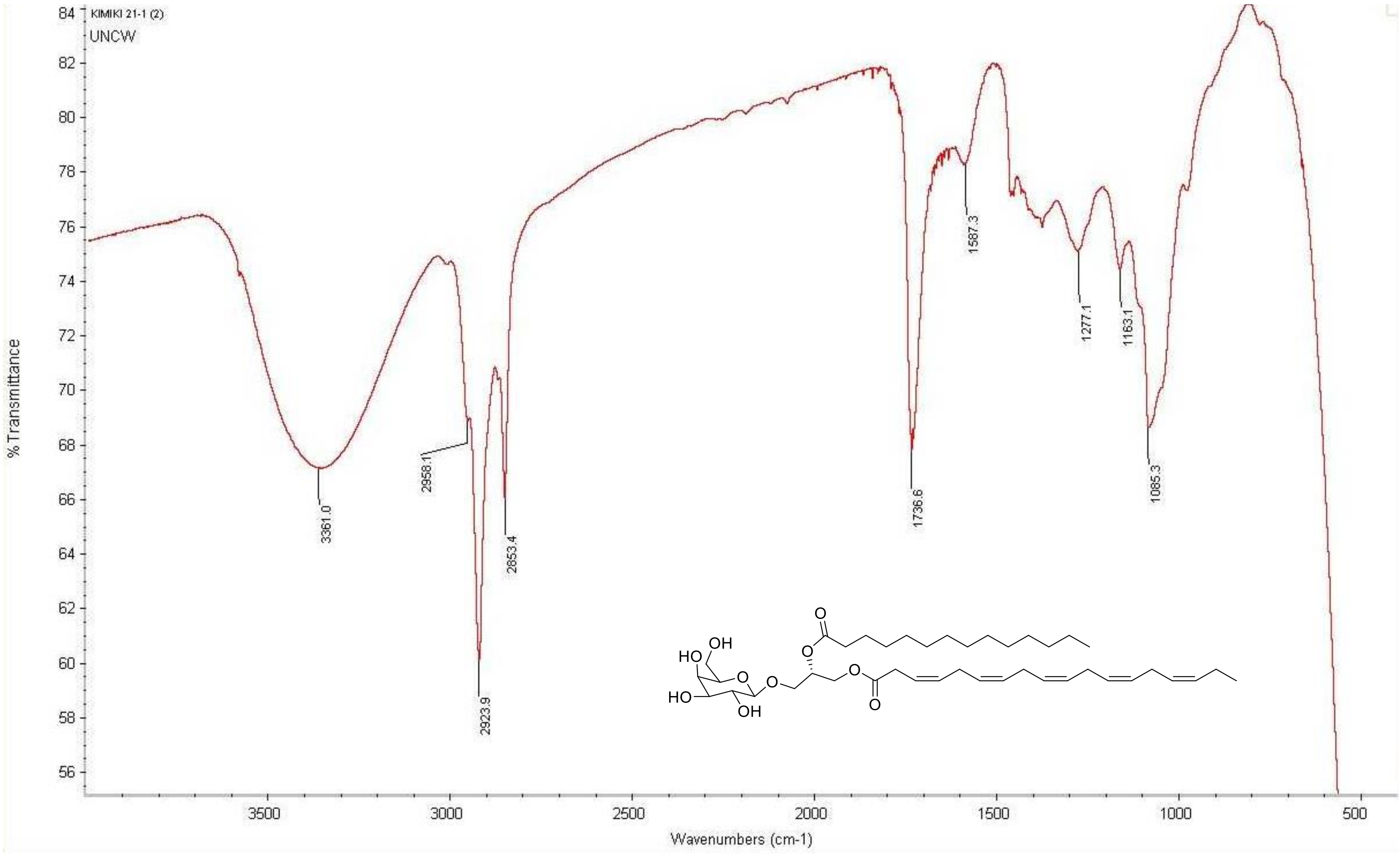

**Figure S9.** Fragmentation ESI-MS of the monogalactosyldiacylglycerol (**1**)

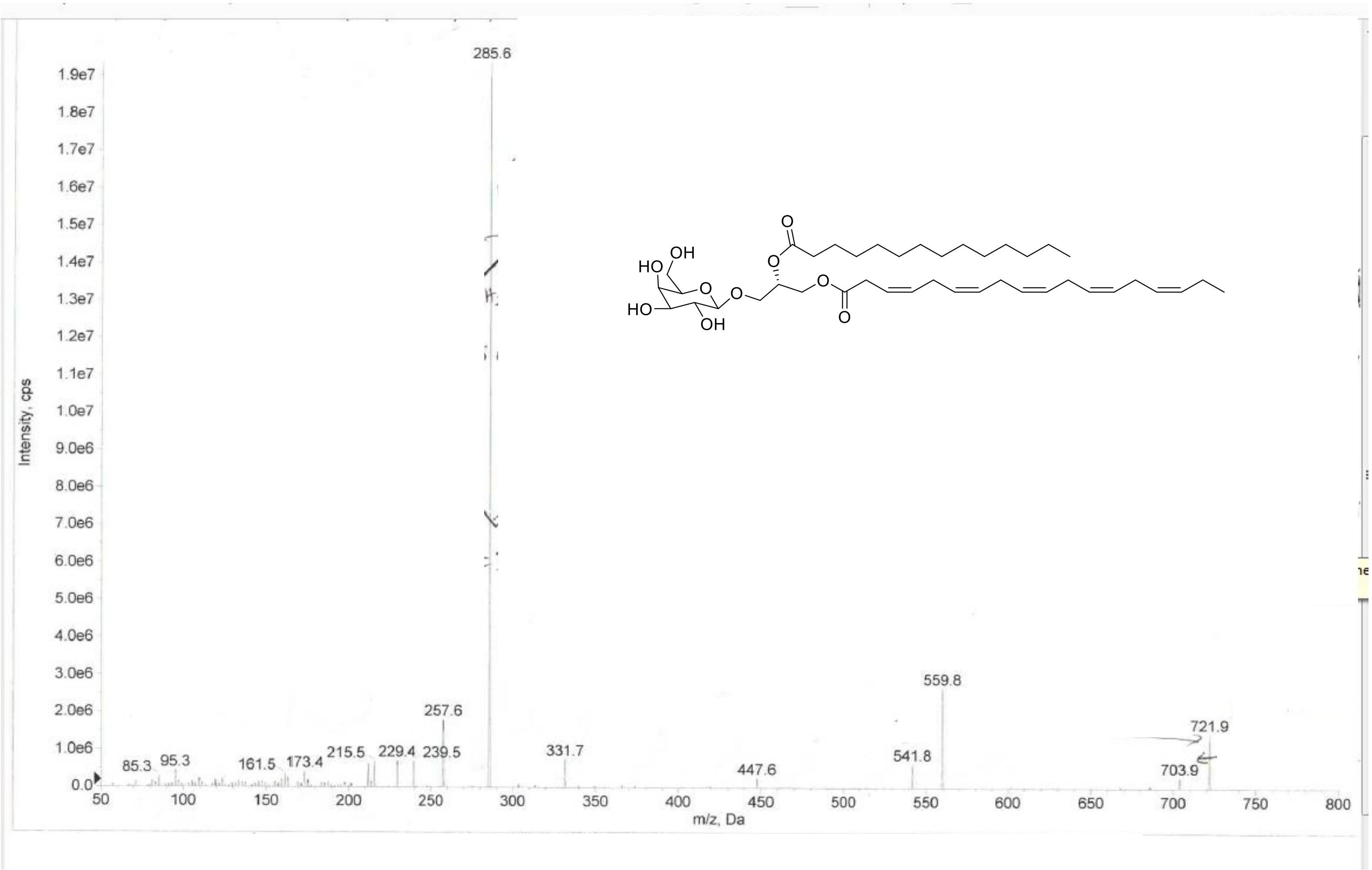

**Figure S10.** $^{1}$H NMR Spectrum (500 MHz) of Monogalactosylmonoacylglycerol (**2**) in $CD_3OD$

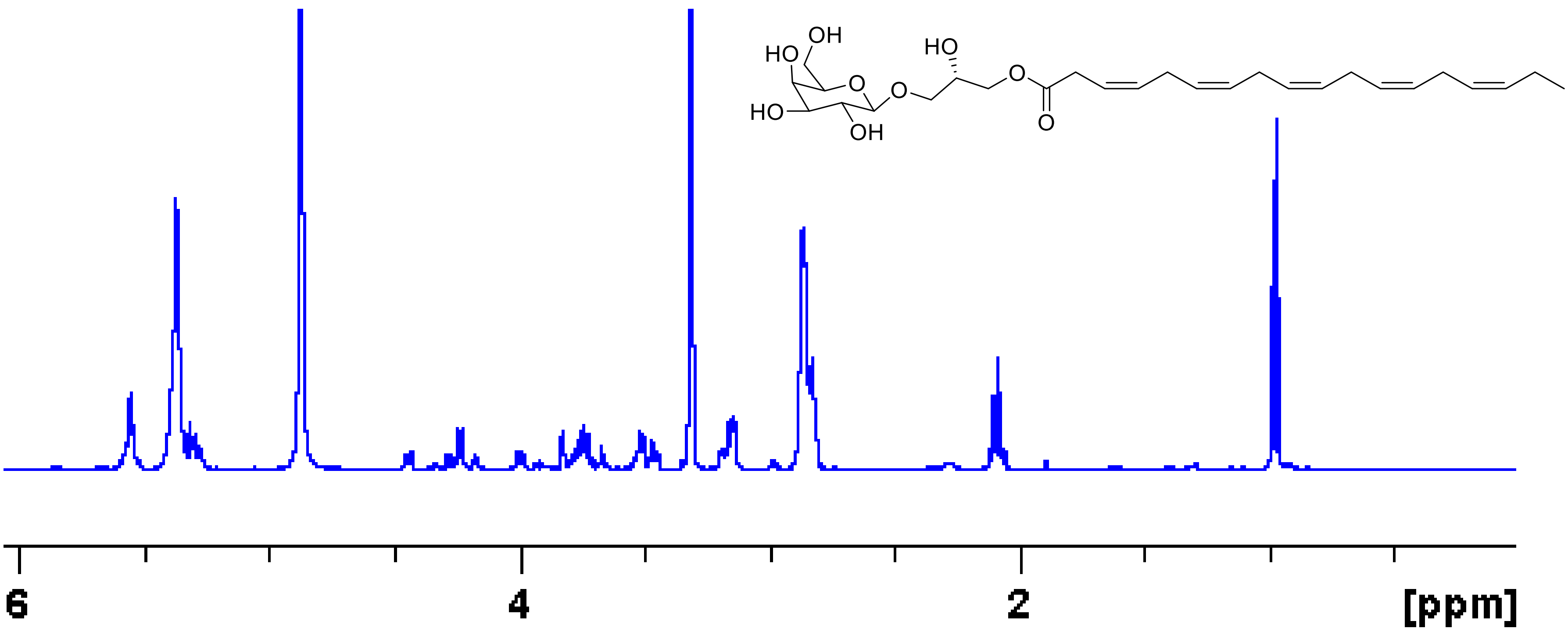

**Figure S11.** $^{13}$C NMR Spectrum (125 MHz) of Monogalactosylmonoacylglycerol **(2)** in $CD_3OD$

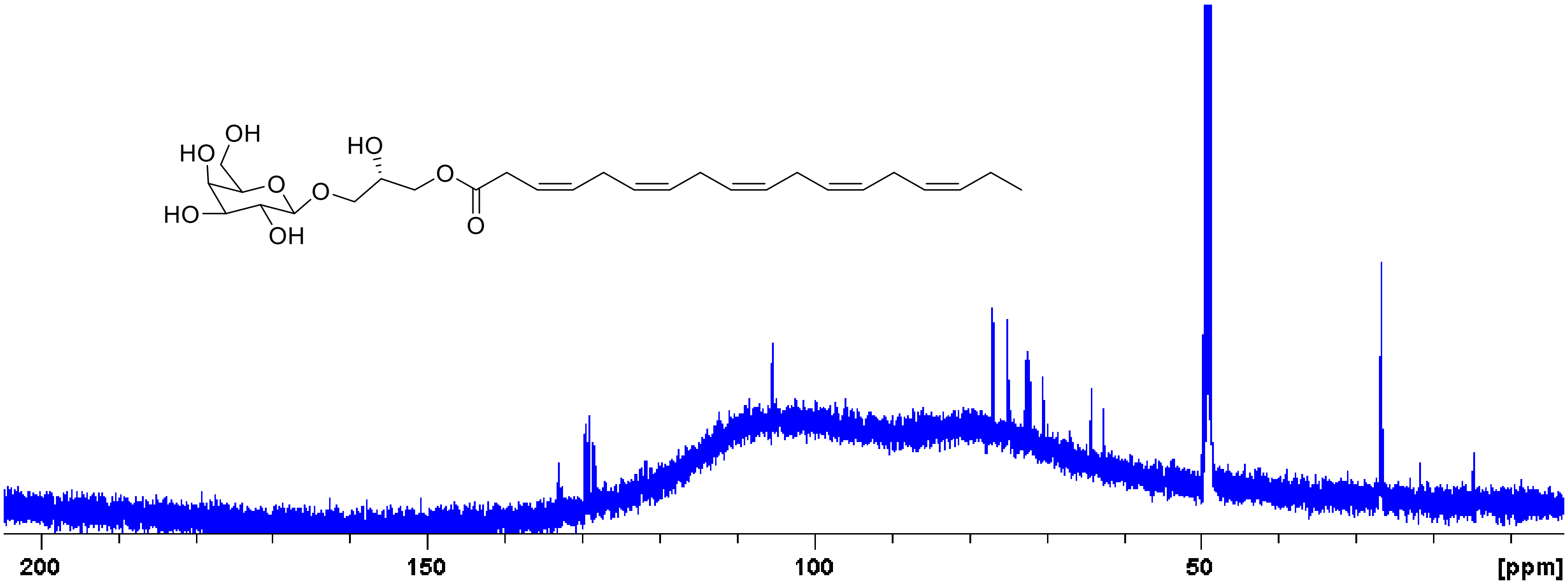

**Figure S12.** HRMSESITOF of Monogalactosylmonoacylglycerol **(2)**

**Single Mass Analysis**
Tolerance = 4.0 PPM / DBE: min = 0.0, max = 50.0
Element prediction: Off
Number of isotope peaks used for i-FIT = 3
Monoisotopic Mass, Odd and Even Electron Ions
208 formula(e) evaluated with 1 results within limits (all results (up to 1000) for each mass)
Elements Used:

| Mass | Calc. Mass | mDa | PPM | DBE | Formula | i-FIT | i-FIT Norm | Fit Conf % | C | H | O | Na |
|---|---|---|---|---|---|---|---|---|---|---|---|---|
| 533.2727 | 533.2727 | 0.0 | 0.0 | 6.5 | C27 H42 O9 Na | 276.3 | n/a | n/a | 27 | 42 | 9 | 1 |

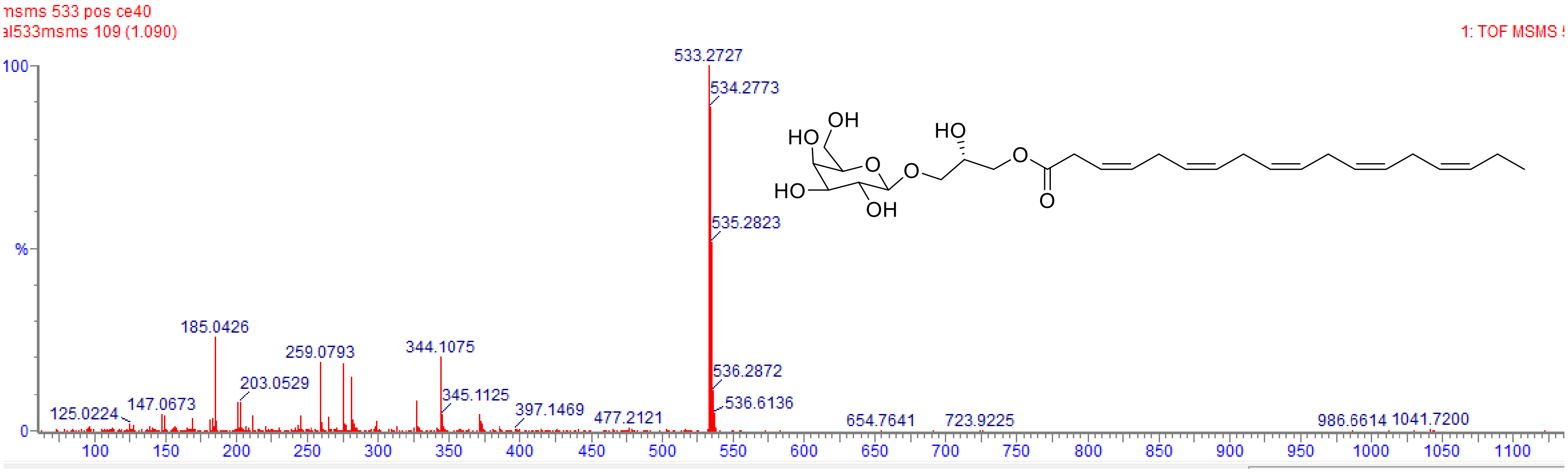

**Figure S13.** $^{1}$H NMR Spectrum (500 MHz) of Polyunsaturated fatty acid methyl ester (**3**) in $CD_3OD$

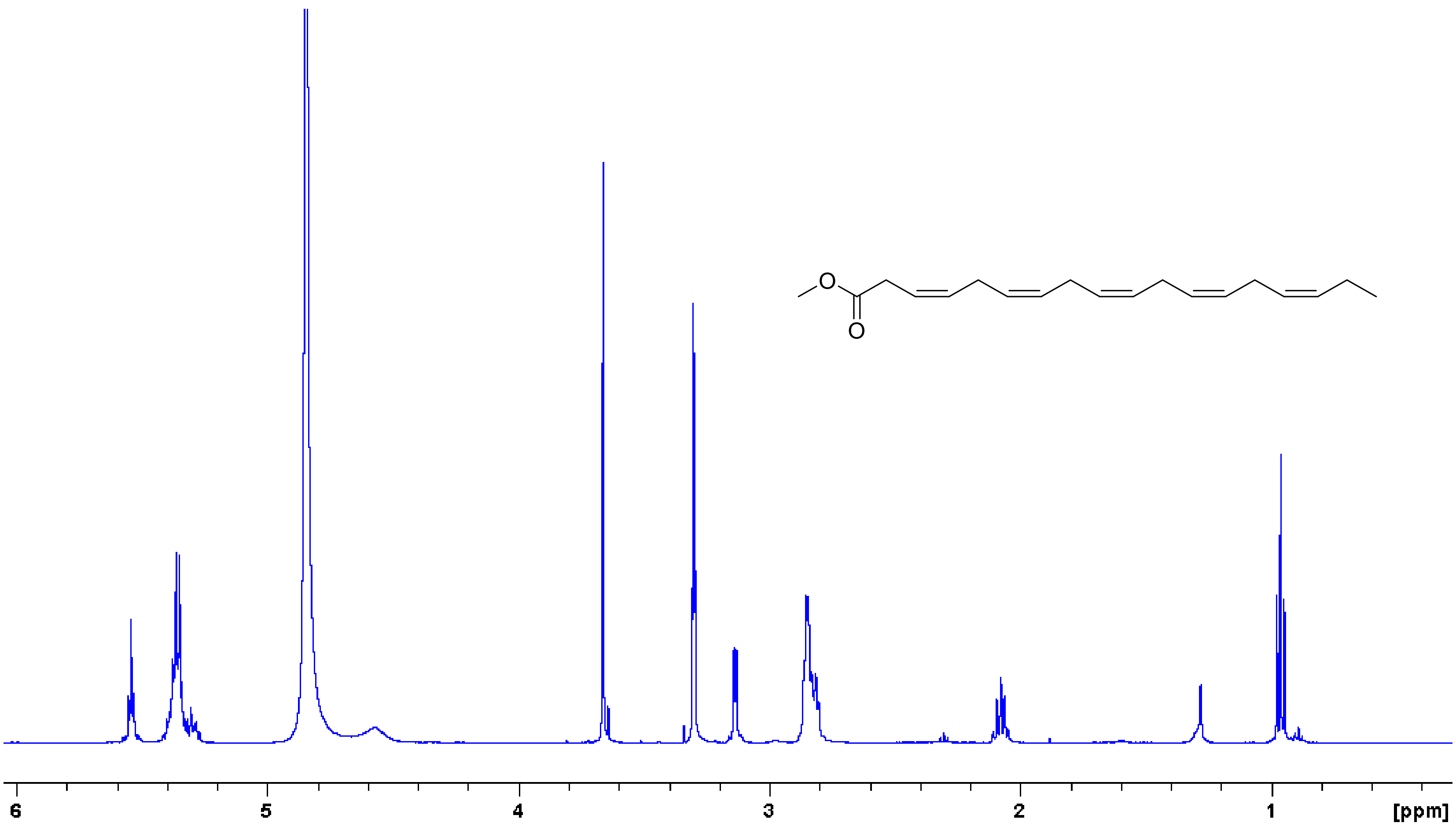

**Figure S14.** $^{13}$C NMR Spectrum (125 MHz) of Polyunsaturated fatty acid methyl ester (**3**) in $CD_3OD$

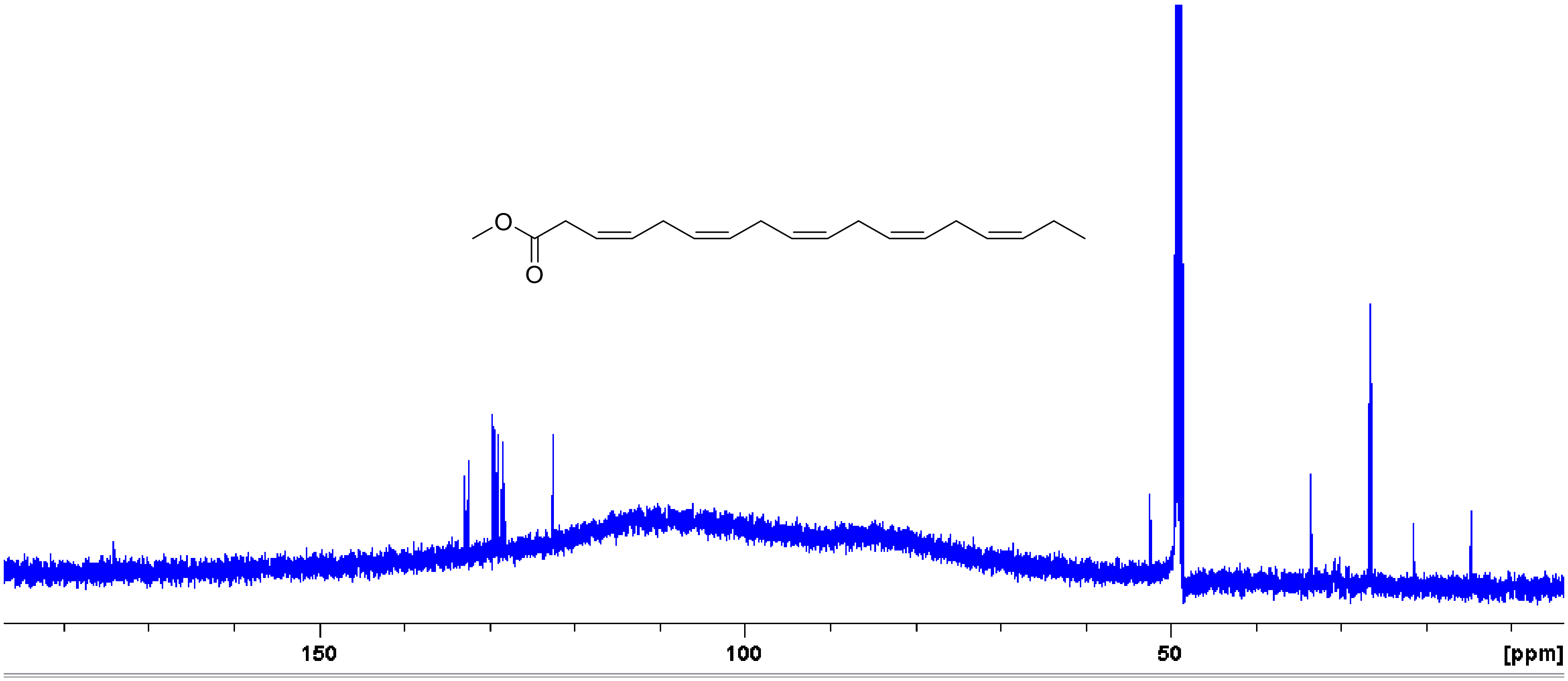

**Figure S15.** HRMSESITOF of Polyunsaturated fatty acid methyl ester (**3**)

**Single Mass Analysis**
Tolerance = 4.0 PPM / DBE: min = 0.0, max = 50.0
Element prediction: Off
Number of isotope peaks used for i-FIT = 3
Monoisotopic Mass, Even Electron Ions
84 formula(e) evaluated with 1 results within limits (all results (up to 1000) for each mass)
Elements Used:

| Mass | Calc. Mass | mDa | PPM | DBE | Formula | i-FIT | i-FIT Norm | Fit Conf % | C | H | O | Na |
|---|---|---|---|---|---|---|---|---|---|---|---|---|
| 289.2169 | 289.2168 | 0.1 | 0.3 | 5.5 | C19 H29 O2 | 815.8 | n/a | n/a | 19 | 29 | 2 | |

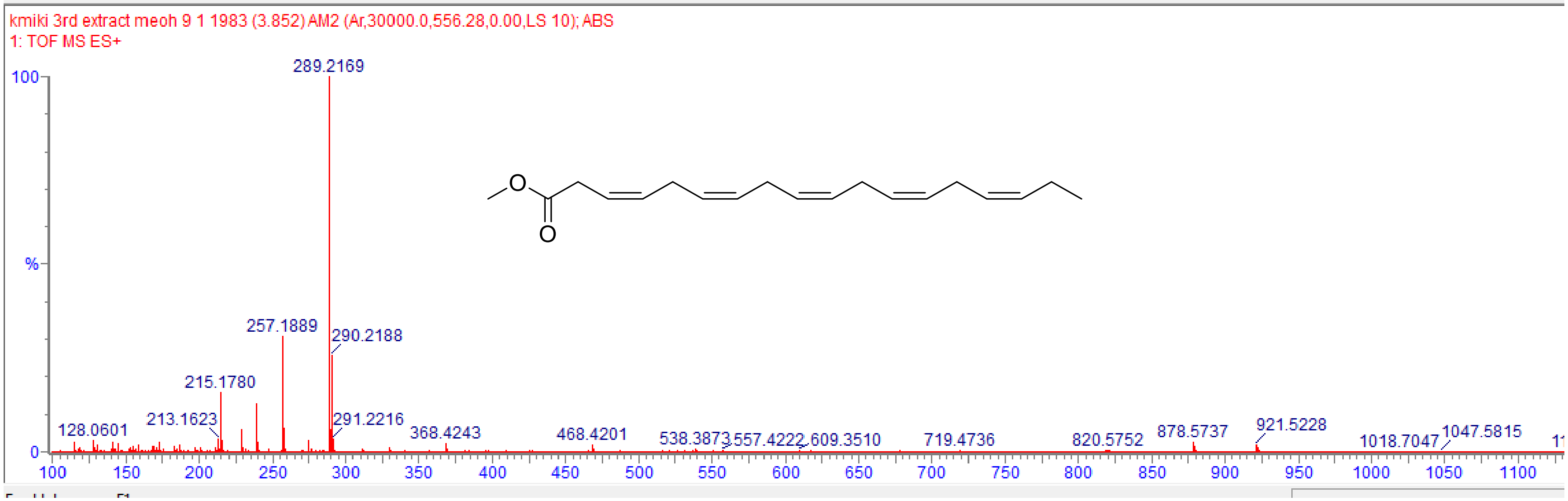

Supplement: Supplementary file 1 [file marinedrugs-18-00138-s001.pdf]
